# Supplementary figures and images for: Genomic assessment reveals signal of adaptive selection in populations of the Spotted rose snapper Lutjanus guttatus from the Tropical Eastern Pacific
Source: PeerJ. 2023 Mar 27;11:e15029. doi: 10.7717/peerj.15029 (PMC10062342; doi:10.7717/peerj.15029)

A) Index of Association  
Neutral loci

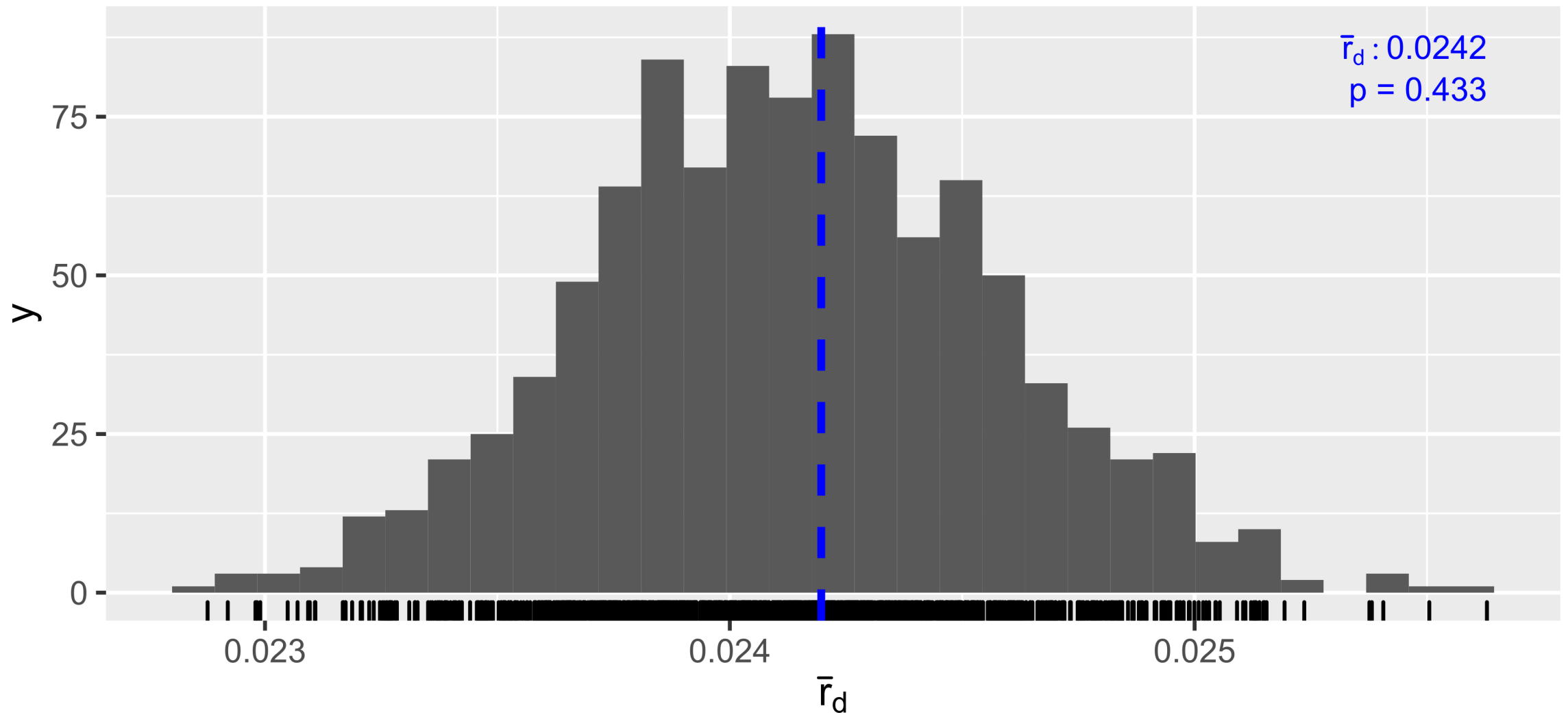

B) Index of Association  
Outlier loci

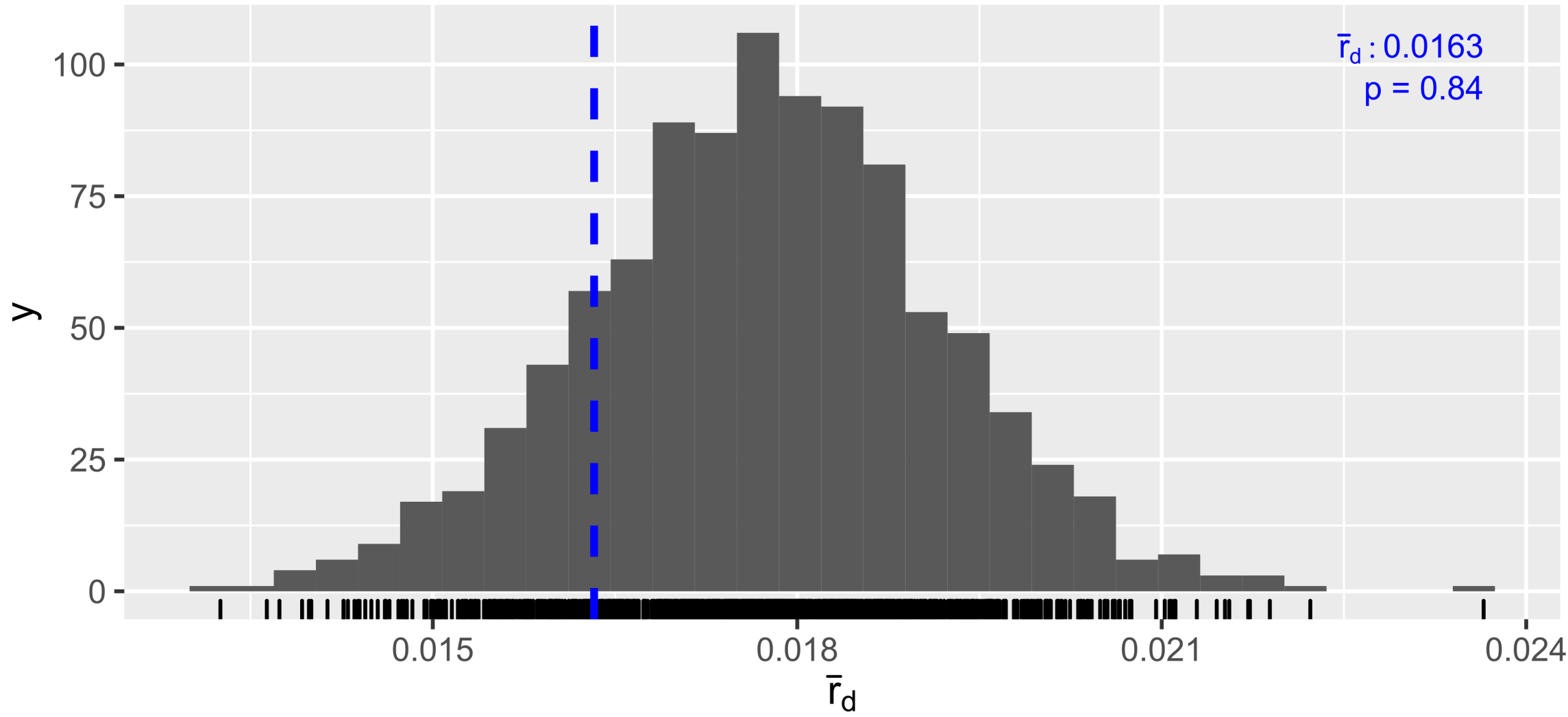

Supplement: Figure S1 — Index of Association calculated for (A) NL and (B) OL datasets (1858 and 145 SNPs, respectively). [file peerj-11-15029-s001.pdf]

## NEUTRAL LOCI

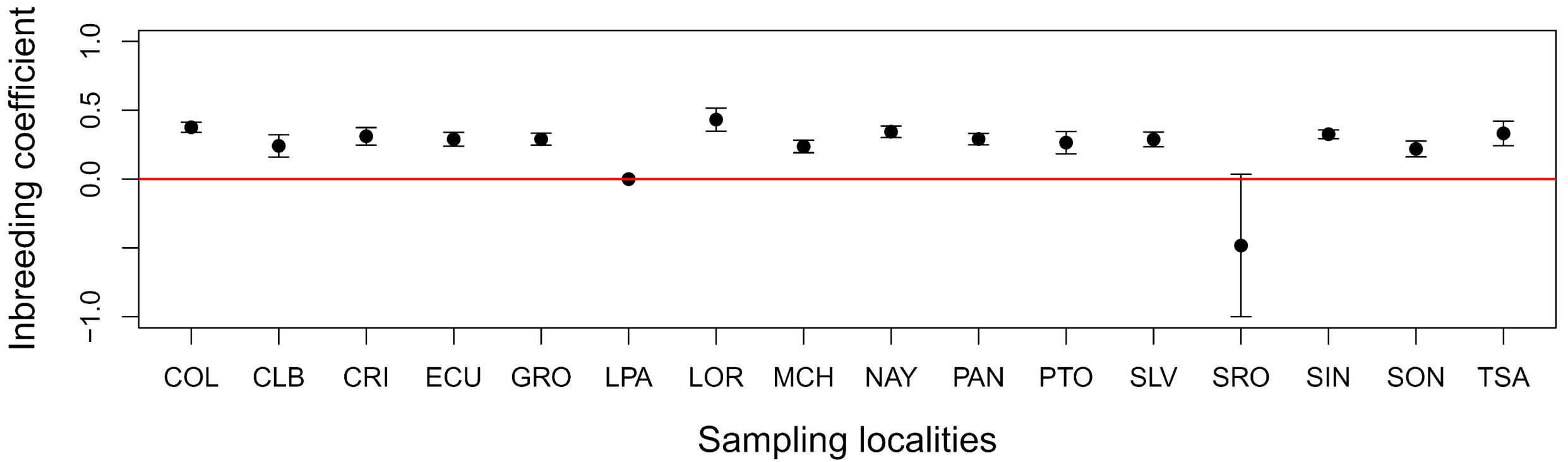

## OUTLIER LOCI

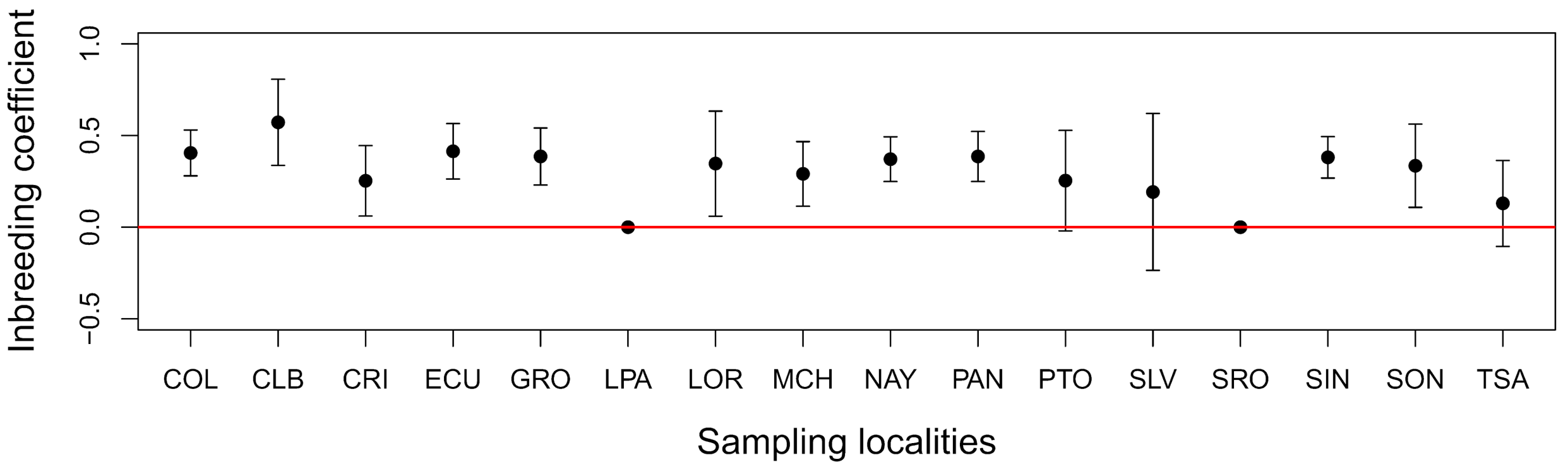

Supplement: Figure S2 — Confidence values for the inbreeding coefficient at 95% confidence intervals for each locality. [file peerj-11-15029-s002.pdf]

**A) NEUTRAL LOCI**

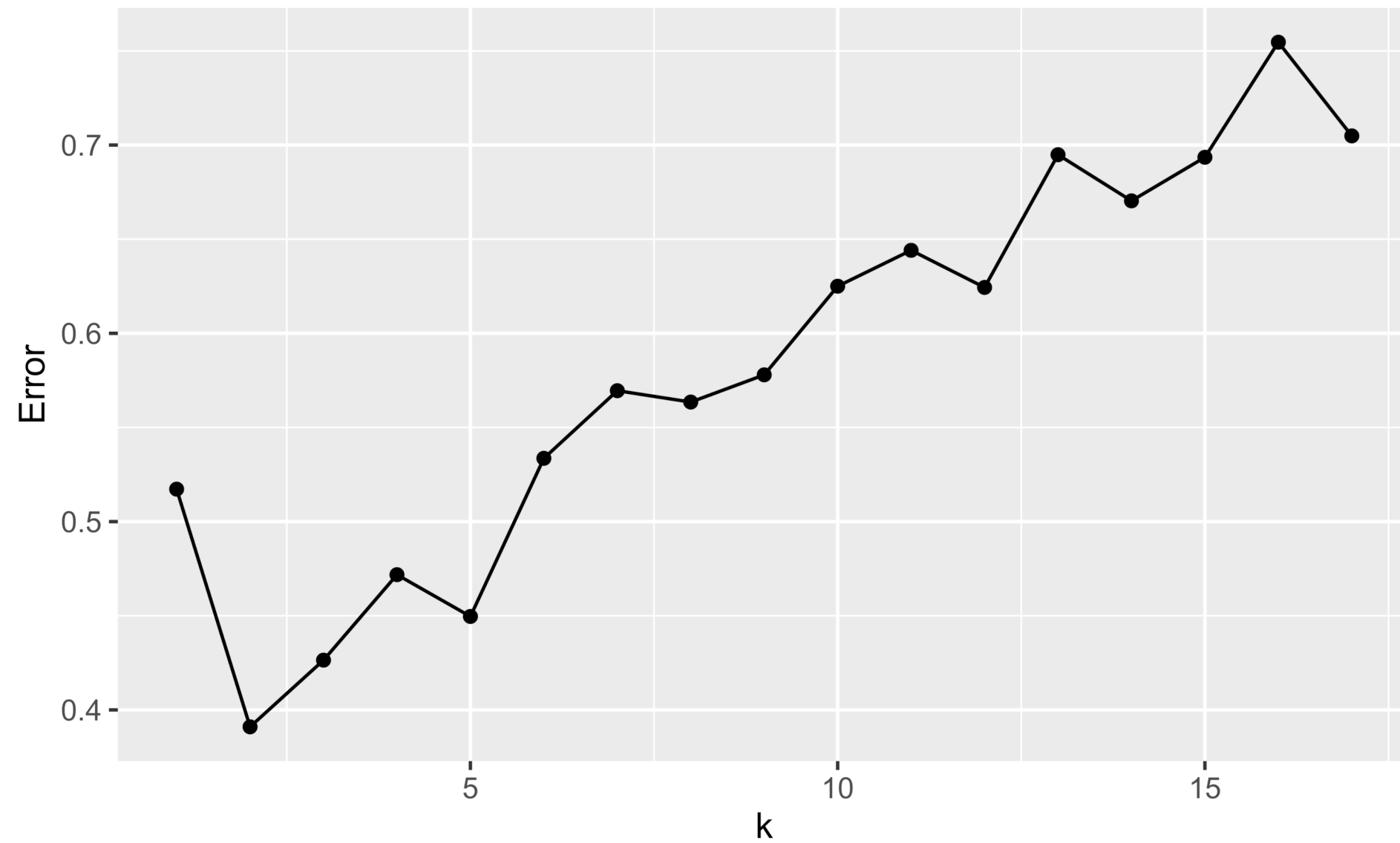

**B) OUTLIER LOCI**

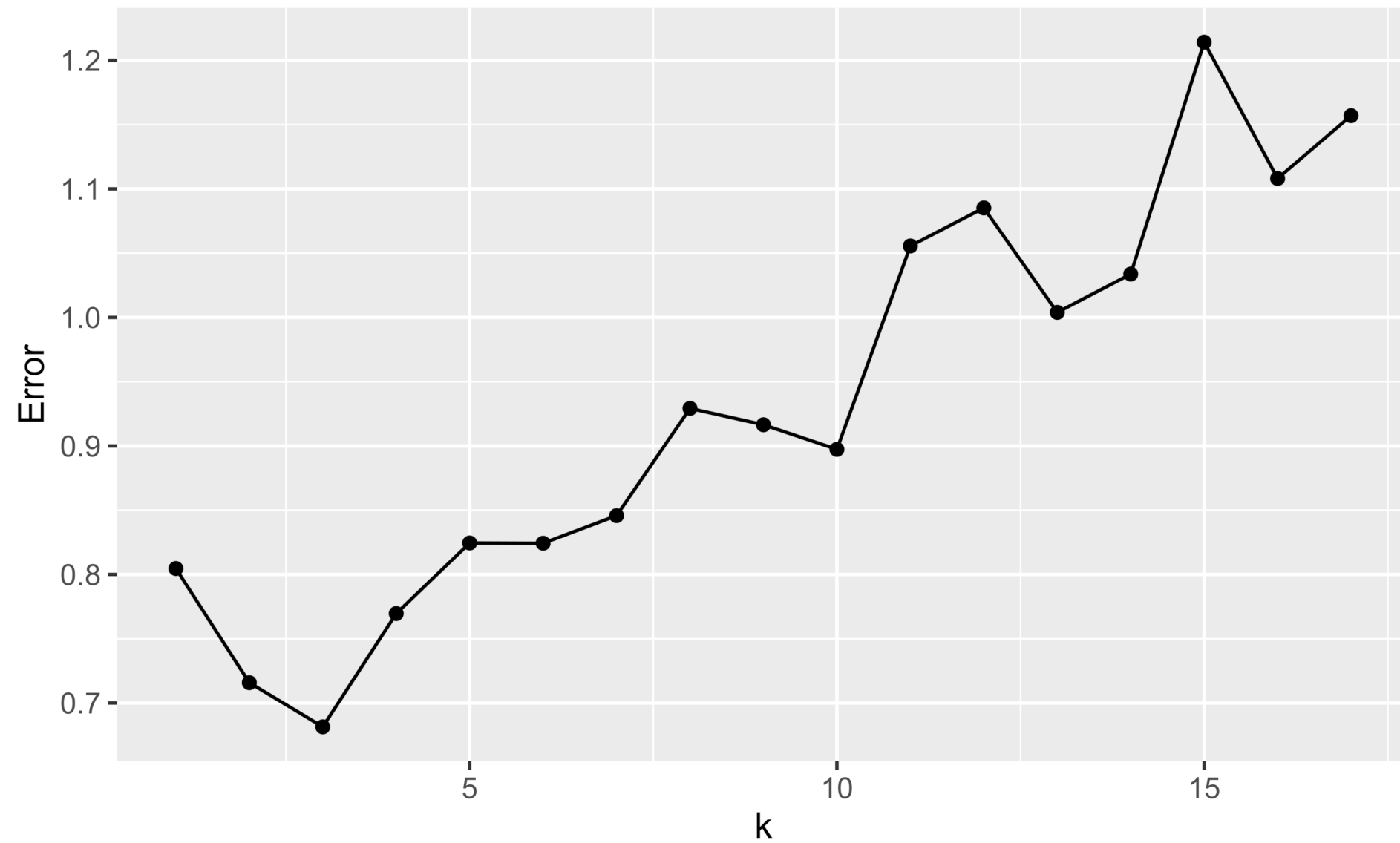

Supplement: Figure S3 — Evaluation of the number of clusters obtained with ADMIXTURE using (A) NL and (B) OL datasets (1858 and 145 SNPs, respectively). [file peerj-11-15029-s003.pdf]

## A) IBD NEUTRAL LOCI

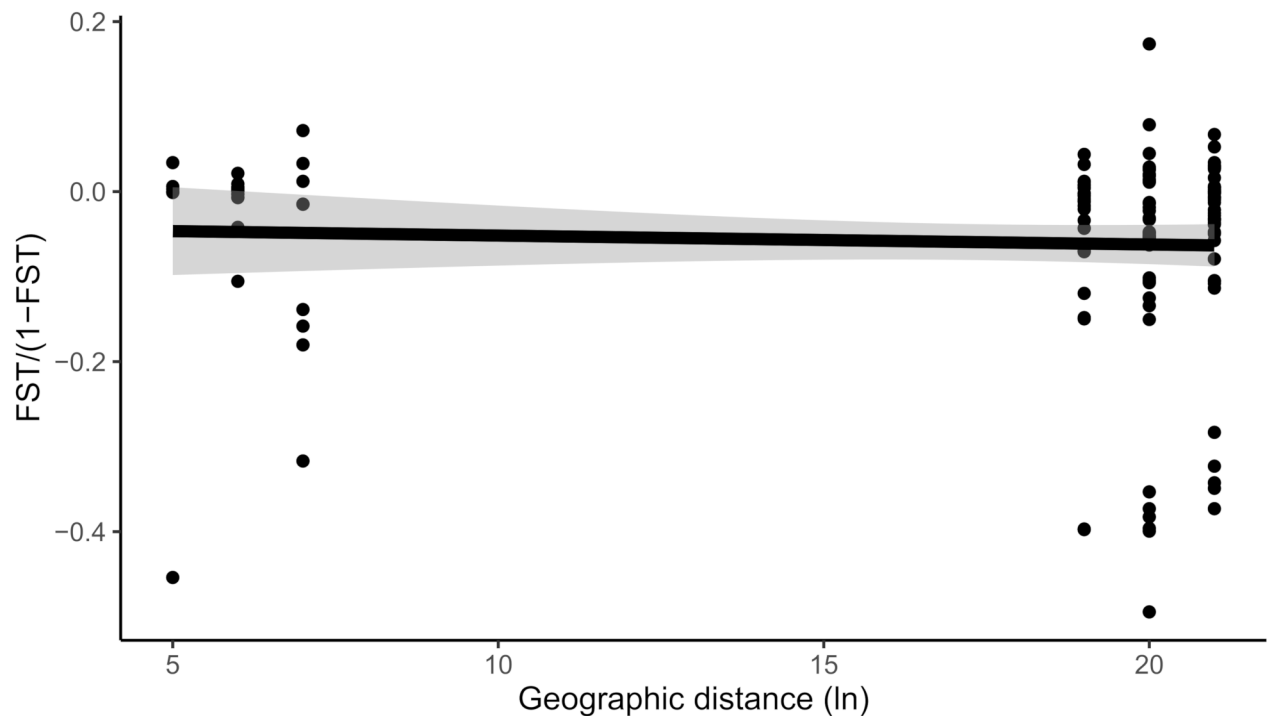

**$r = 0.0587$**   
 **$p = 0.278$**

## B) IBD OUTLIER LOCI

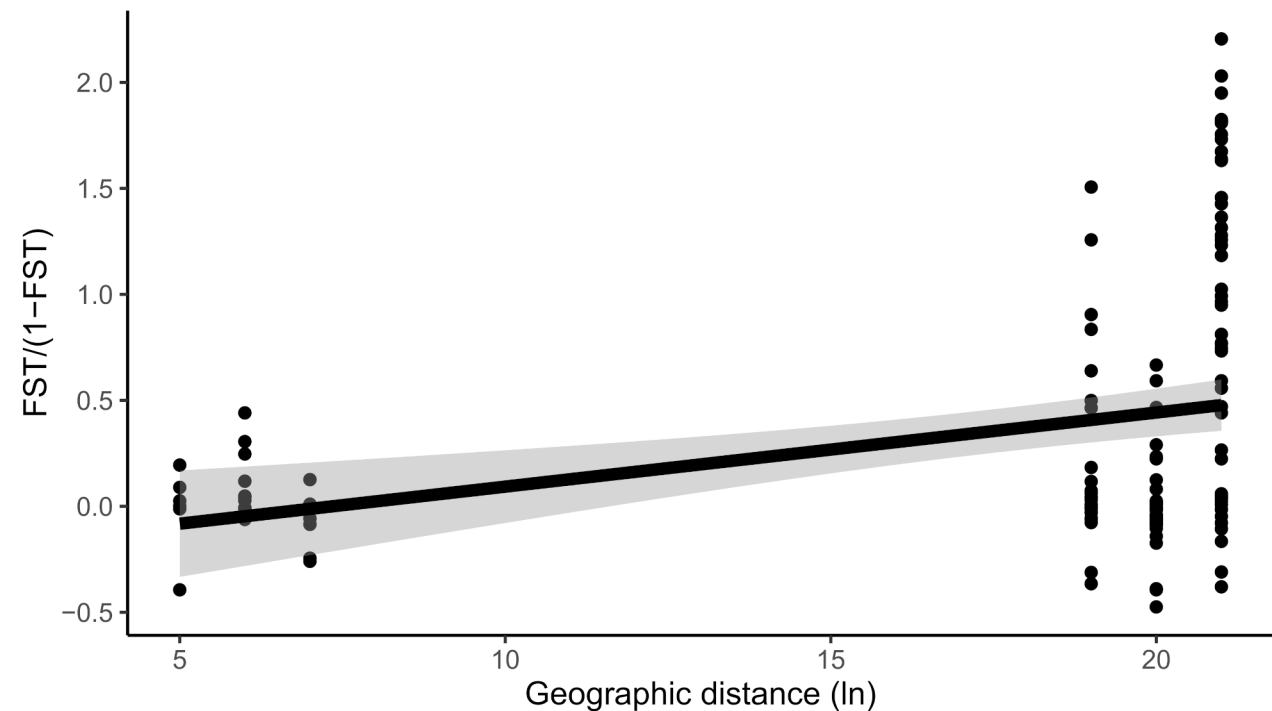

**$r = 0.0970$**   
 **$p = 0.174$**

Supplement: Figure S4 — Mantel test for Lutjanus guttatus using (A) NL and (B) OL datasets (1858 and 145 SNPs, respectively). Geographic distances were transformed to natural logarithm (ln). [file peerj-11-15029-s004.pdf]

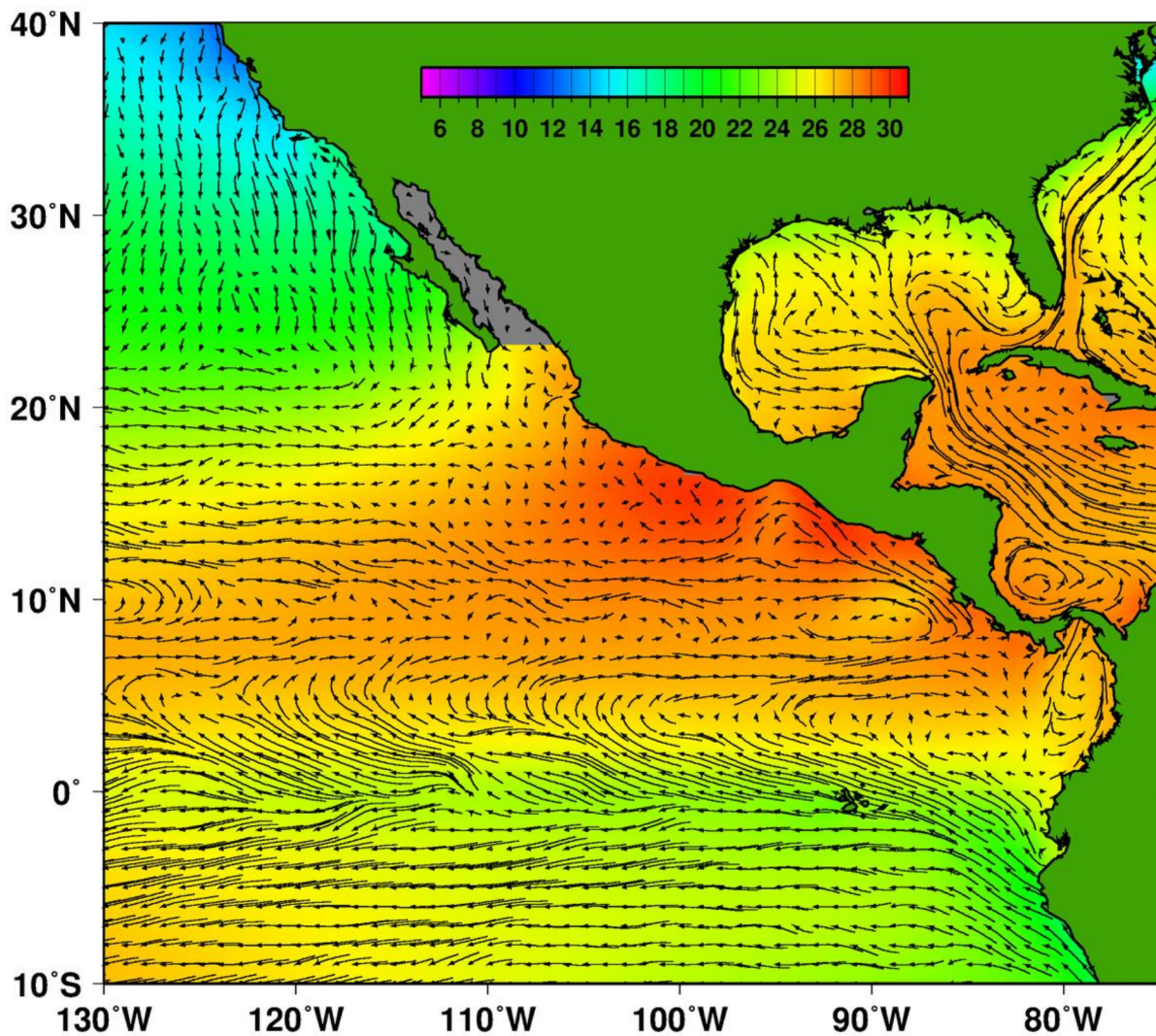

Supplement: Figure S6 — Map of the oceanographic currents of the tropical Eastern pacific (TEP). Map is based on Mariano, A.J. and E.H. Ryan, 2018. http://oceancurrents.rsmas.miami.edu/. Scale bar is in °C; values shown represent long term average temperature records for April. [file peerj-11-15029-s006.pdf]

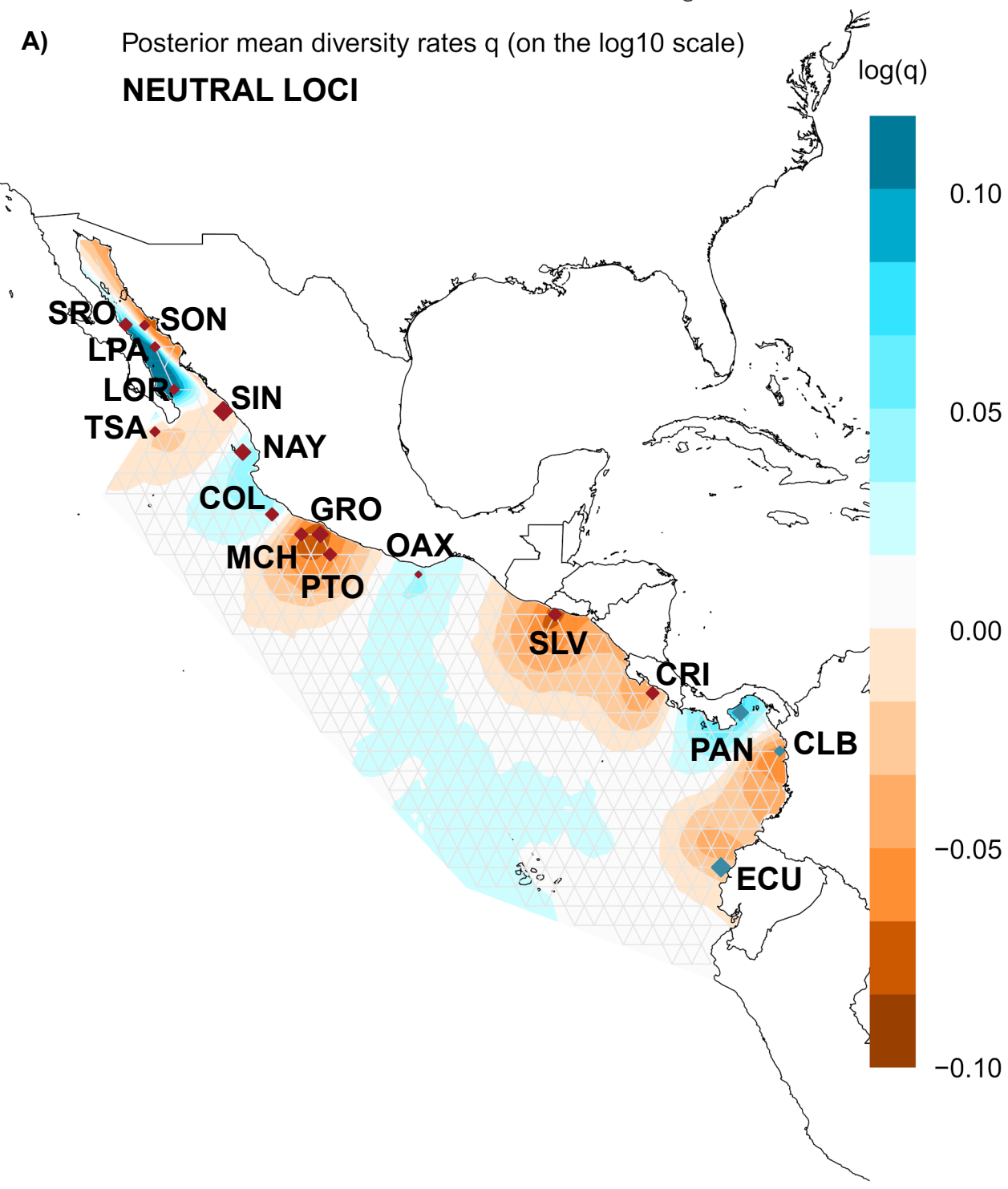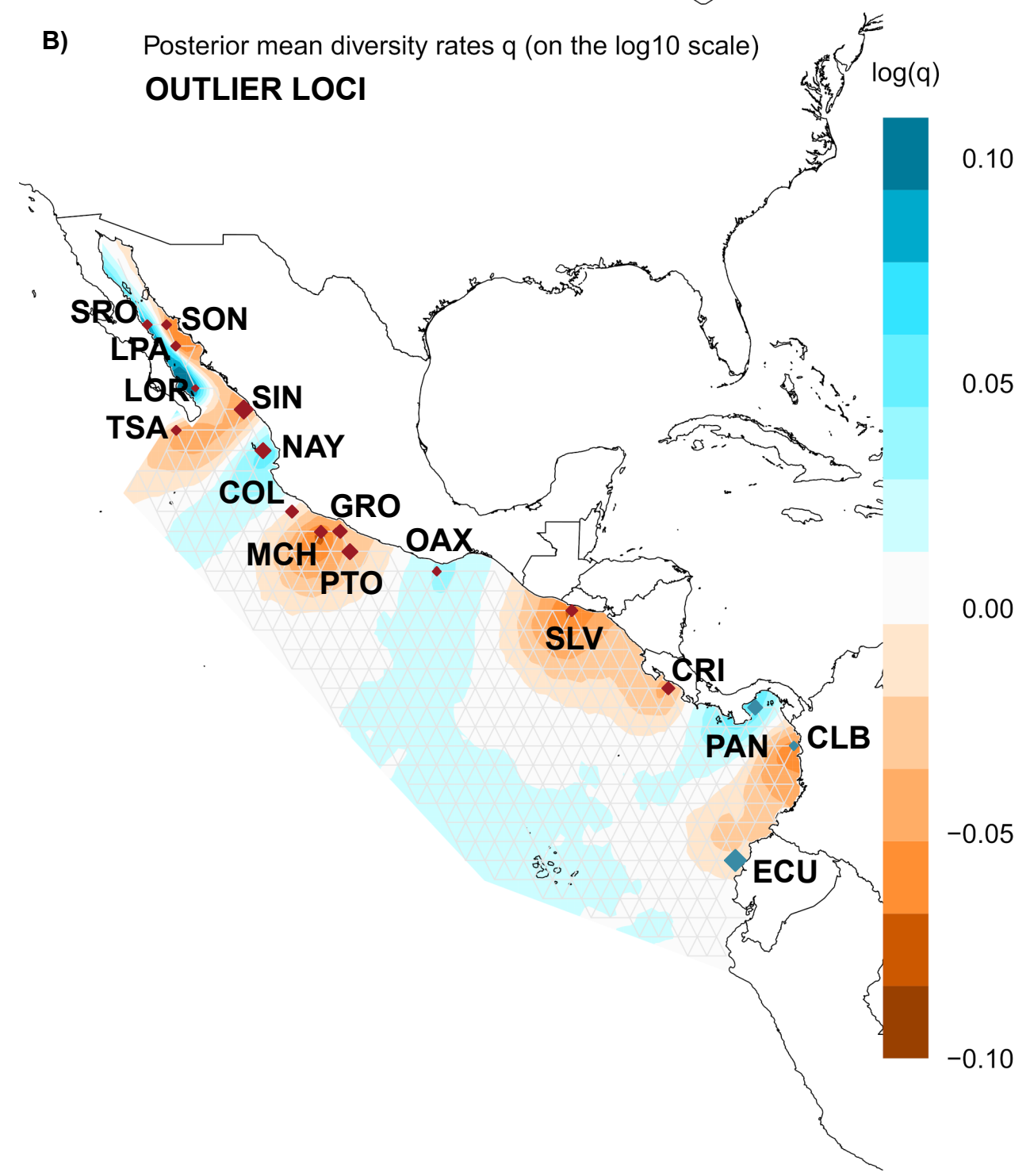

Supplement: Figure S7 — Results for the posterior mean diversity rates (q), obtained with estimating effective migration surfaces (EEMS) analysis using (A) NL and (B) OL datasets (1858 and 145 SNPs, respectively). Higher values than average genetic diversity are presented in blue, while lower values are depicted in brown. [file peerj-11-15029-s007.pdf]
