# Supplementary material for: Genomic assessment reveals signal of adaptive selection in populations of the Spotted rose snapper Lutjanus guttatus from the Tropical Eastern Pacific
Source: PeerJ. 2023 Mar 27;11:e15029. doi: 10.7717/peerj.15029 (PMC10062342; doi:10.7717/peerj.15029)
Supplement: Figure S5 — Mantel test for Northern and Southern groups for Lutjanus guttatus using NL dataset (A) and (C) 1858 SNPs and OL dataset (B) and (D) 145 SNPs. [file peerj-11-15029-s005.pdf]

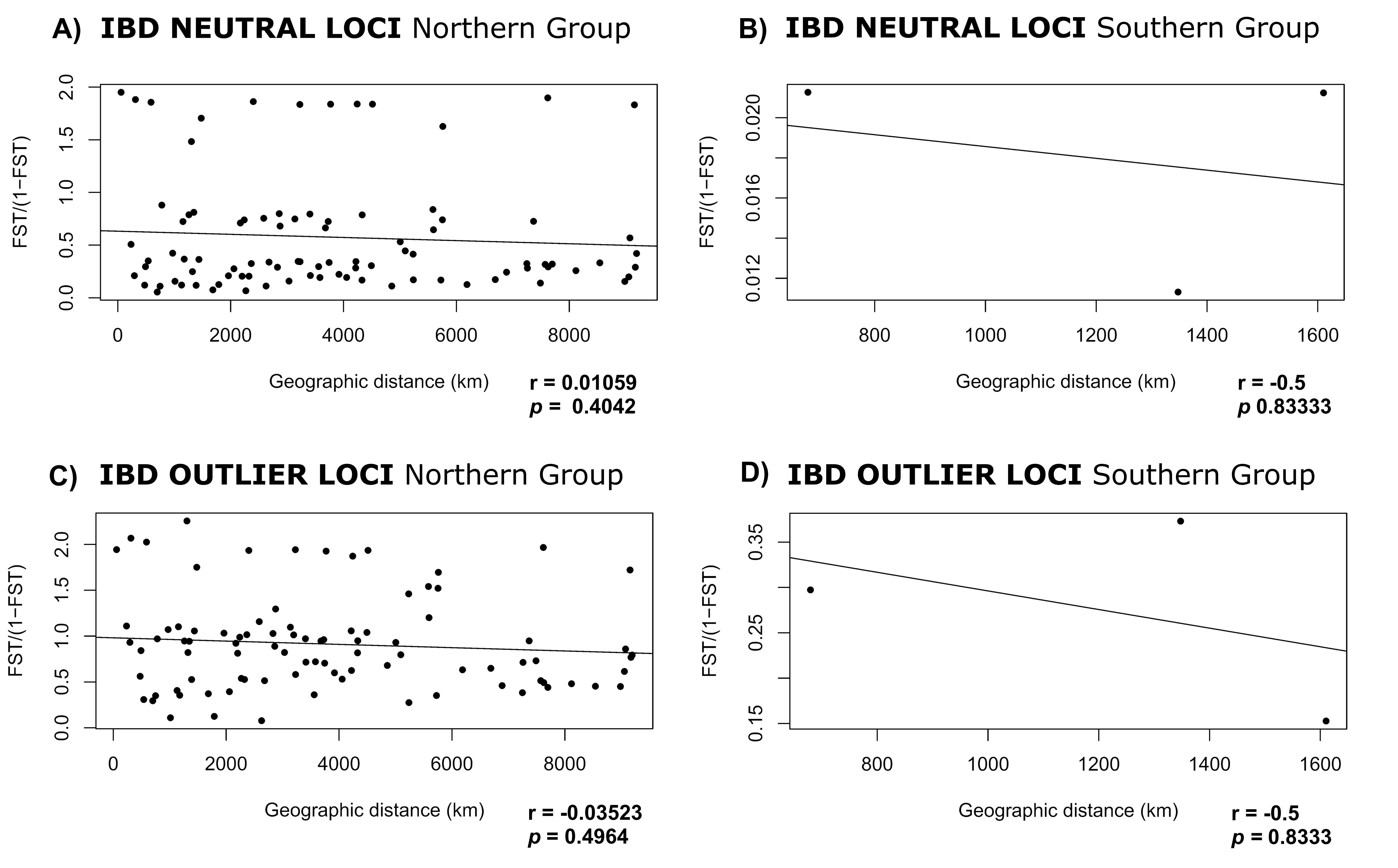

A) IBD NEUTRAL LOCI Northern Group
FST/(1−FST)
Geographic distance (km)
r = 0.01059
p = 0.4042
B) IBD NEUTRAL LOCI Southern Group
FST/(1−FST)
Geographic distance (km)
r = -0.5
p 0.83333
C) IBD OUTLIER LOCI Northern Group
FST/(1−FST)
Geographic distance (km)
r = -0.03523
p = 0.4964
D) IBD OUTLIER LOCI Southern Group
FST/(1−FST)
Geographic distance (km)
r = -0.5
p = 0.8333
