# Supplementary material for: Genomic assessment reveals signal of adaptive selection in populations of the Spotted rose snapper Lutjanus guttatus from the Tropical Eastern Pacific
Source: PeerJ. 2023 Mar 27;11:e15029. doi: 10.7717/peerj.15029 (PMC10062342; doi:10.7717/peerj.15029)
Supplement: Table S1 — Final dataset of 123 samples retained after the second filters (minor allele frequency maf = 0.01, percentage of missing data 20%). [file peerj-11-15029-s008.docx]

**Table S1. Samples of *Lutjanus guttatus* filtered using *VCFT*ools**. Final dataset of 123 samples retained after the second filters (minor allele frequency maf = 0.01, percentage of missing data 20%).

| **Sample** | **Code** | **Group** |
| --- | --- | --- |
| SR4 | SRO | Northern |
| SR9 | SRO | Northern |
| SR15 | SRO | Northern |
| Son10 | SON | Northern |
| Son11 | SON | Northern |
| Son14 | SON | Northern |
| Son15 | SON | Northern |
| Son19 | SON | Northern |
| Lore1 | LOR | Northern |
| Loreto3 | LOR | Northern |
| Lore4 | LOR | Northern |
| LPaz2 | LPA | Northern |
| LPaz4 | LPA | Northern |
| TS13 | TSA | Northern |
| TS17 | TSA | Northern |
| TS18 | TSA | Northern |
| TS21 | TSA | Northern |
| TS30 | TSA | Northern |
| Mz9 | SIN | Northern |
| Mz11 | SIN | Northern |
| Mz12 | SIN | Northern |
| Mz13 | SIN | Northern |
| Mz15 | SIN | Northern |
| Mz3 | SIN | Northern |
| Mz44 | SIN | Northern |
| Mz49 | SIN | Northern |
| Mz5 | SIN | Northern |
| Mz50 | SIN | Northern |
| Mz58 | SIN | Northern |
| Mz59 | SIN | Northern |
| Mz60 | SIN | Northern |
| Mz7 | SIN | Northern |
| Mz8 | SIN | Northern |
| BB9 | NAY | Northern |
| BB15 | NAY | Northern |
| BB16 | NAY | Northern |
| BB19 | NAY | Northern |
| BB20 | NAY | Northern |
| BB21 | NAY | Northern |
| BB22 | NAY | Northern |
| BB23 | NAY | Northern |
| BB25 | NAY | Northern |
| BB27 | NAY | Northern |
| BB29 | NAY | Northern |
| BB8 | NAY | Northern |
| L3 | COL | Northern |
| L7 | COL | Northern |
| L9 | COL | Northern |
| L10 | COL | Northern |
| L14 | COL | Northern |
| L16 | COL | Northern |
| L17 | COL | Northern |
| L21 | COL | Northern |
| L25 | COL | Northern |
| Mich1 | MCH | Northern |
| Mich4 | MCH | Northern |
| Mich5 | MCH | Northern |
| Mich8 | MCH | Northern |
| Mich9 | MCH | Northern |
| Mich14 | MCH | Northern |
| Mich2 | MCH | Northern |
| Mich6 | MCH | Northern |
| Gro2 | GRO | Northern |
| Gro3 | GRO | Northern |
| Gro4 | GRO | Northern |
| Gro5 | GRO | Northern |
| Gro9 | GRO | Northern |
| Gro10 | GRO | Northern |
| Gro11 | GRO | Northern |
| Gro12 | GRO | Northern |
| Gro14 | GRO | Northern |
| Gro8 | GRO | Northern |
| Pr1 | PTO | Northern |
| Pr2 | PTO | Northern |
| Pr3 | PTO | Northern |
| Oax4 | OAX | Northern |
| Salv9 | SLV | Northern |
| Salv10 | SLV | Northern |
| Salv20 | SLV | Northern |
| Salv22 | SLV | Northern |
| Salv55 | SLV | Northern |
| Salv56 | SLV | Northern |
| Salv57 | SLV | Northern |
| Salv59 | SLV | Northern |
| CR1 | CRI | Northern |
| CR4 | CRI | Northern |
| CR8 | CRI | Northern |
| CR11 | CRI | Northern |
| CR12 | CRI | Northern |
| CR13 | CRI | Northern |
| CR14 | CRI | Northern |
| CR15 | CRI | Northern |
| cr10 | CRI | Northern |
| Pan5 | PAN | Southern |
| Pan11 | PAN | Southern |
| Pan12 | PAN | Southern |
| Pan19 | PAN | Southern |
| Pan26 | PAN | Southern |
| Pan27 | PAN | Southern |
| Pan32 | PAN | Southern |
| Pan34 | PAN | Southern |
| Pan36 | PAN | Southern |
| Pan38 | PAN | Southern |
| Pan39 | PAN | Southern |
| Pan41 | PAN | Southern |
| Cho10 | CLB | Southern |
| Cho5 | CLB | Southern |
| Cho6 | CLB | Southern |
| cho8 | CLB | Southern |
| Cho9 | CLB | Southern |
| Ecu1 | ECU | Southern |
| Ecu3 | ECU | Southern |
| Ecu5 | ECU | Southern |
| Ecu7 | ECU | Southern |
| Mana1 | ECU | Southern |
| Mana2 | ECU | Southern |
| Mana4 | ECU | Southern |
| Mana5 | ECU | Southern |
| Mana6 | ECU | Southern |
| Mana7 | ECU | Southern |
| Mana8 | ECU | Southern |
| Mana9 | ECU | Southern |
| Mana10 | ECU | Southern |
