# Supplementary material for: Genomic assessment reveals signal of adaptive selection in populations of the Spotted rose snapper Lutjanus guttatus from the Tropical Eastern Pacific
Source: PeerJ. 2023 Mar 27;11:e15029. doi: 10.7717/peerj.15029 (PMC10062342; doi:10.7717/peerj.15029)
Supplement: Table S2 — Results of SAMOVA for the NL and OL datasets (1858 and 145 SNPs, respectively). The best representation of the observed clusters was obtained without previously defined groups. [file peerj-11-15029-s009.docx]

**Table S2. Results of SAMOVA for the NL and OL datasets (1858 and 145 SNPs, respectively).** The best representation of the observed clusters was obtained without previously defined groups.

| **NL** | | | | |
| --- | --- | --- | --- | --- |
| **Two groups** | **Source of varation** | **% of variance** | **Fixation index** | ***p*- value** |
| [SRO-SON-LOR-LPA-TSA-SIN-NAY-COL-MCH-GRO-PTO-OAX-SLV-CRI-PAN-ECU]  +  [CLB] | Among groups | 3.43 | F_CT_ = 0.0342 | NS |
|  | Among populations within groups | -2.95 | F_SC_ = -0.0305 | NS |
|  | Among individuals within population | 5.15 | F_IS_ = 0.0517 | 0.001 |
|  | Within populations | 94.37 | F_ST_ = 0.0562 | 0.03 |
| **OL** | | | | |
| **Two groups** | **Source of varation** | **% of variance** | **Fixation index** | ***p*- value** |
| [SRO-SON-LOR-LPA-TSA-SIN-NAY-COL-MCH-GRO-PTO-OAX-SLV-CRI]  +  [PAN-CLB-ECU] | Among groups | 48.29 | F_CT_ = 0.4860 | 0.001 |
|  | Among populations within groups | 0.68 | F_SC_ = 0.0270 | NS |
|  | Among individuals within population | -0.28 | F_IS_ = -0.0054 | NS |
|  | Within populations | 51.31 | F_ST_ = 0.4972 | < 0.001 |

**Note:** NS = no significant
