# Supplementary material for: Genomic assessment reveals signal of adaptive selection in populations of the Spotted rose snapper Lutjanus guttatus from the Tropical Eastern Pacific
Source: PeerJ. 2023 Mar 27;11:e15029. doi: 10.7717/peerj.15029 (PMC10062342; doi:10.7717/peerj.15029)
Supplement: File S1 — Identification of polymorphic loci and SR-Snapper-Bait design and synthesis. [file peerj-11-15029-s010.docx]

**File S1. Supplementary information**

**Genomic assessment reveals signal of adaptive selection in populations of the Spotted rose snapper *Lutjanus guttatus* from the Tropical Eastern Pacific**

Adan Fernando Mar-Silva, Pindaro Díaz-Jaimes, Cristina A. Dominguez-Mendoza, Omar Domínguez-Domínguez, Jonathan Valdiviezo-Rivera and Eduardo Espinoza

**Supplementary Material and Methods**

**Identification of polymorphic loci and SR-Snapper-Bait design and synthesis.**

We prepared triple-enzyme RADseq libraries (3RAD), for a total 15 individuals from nine sampling sites across the distribution range of *Lutjanus guttatus* (Table S1), following the protocol of Bayona-Vásquez *et al*. (2019). In brief, DNA was digested using the restriction enzymes *BamHI*, *ClaI*, and *MspI* (New England Biolabs® Inc.), followed by the ligation of adaptors with internal variable length barcodes, and the amplification with iTru-indexed primers (Bayona-Vásquez *et al*., 2019; Glenn *et al.*, 2019). Libraries were pooled in equimolar ratios and size-selected for a 550 bp (+/- 15% range) that were later sequenced on a HiSeq PE150 at the Oklahoma Medical Research Foundation (OK, USA).

The software Stacks *v.* 1.42 (Catchen, *et al*., 2011; Catchen *et al*., 2013) was used to assemble *de novo* the raw sequences by setting the minimum number of reads allowed to form a stack (-*m*) to 3, the number of mismatches between stacks within individuals (-*M*) to 3, and the number of mismatches allowed between stacks between individuals (-*n*) to 4. We generated a catalogue of polymorphic loci present in at least five out of the nine populations (50%) and designed the SR-Snapper-Bait set, which can be used for future studies of the species by using sequence capture (i.e., RADcap, (Hoffberg *et al*., 2016)). The catalogue was stored in a fasta file retaining each allele sequence per individual for only biological plausible loci (--fasta_strict, max two alleles for diploids) We used the program Emboss *v* 6.5.7 (Rice *et al*., 2000), to create two new fasta files, one containing the consensus sequence for each polymorphic locus, where the different alleles that contained single nucleotide polymorphisms (SNPs) were encoded as ambiguities (hereafter refered as the consambig file), and the other file conformed using the same consensus sequence per locus, but instead of ambiguities at polymorphic sites, we retained the base with the highest score (the most frequent) (hereafter referred as the cons file). Finally, we used the R package *Biostrings* (Pagès *et al*., 2019) to count the number of ambiguities in our consambig file, and to determine the number of SNPs per locus for which we filtered out loci with more than five SNPs and a length of < 230 bp to have enough space to potentially synthesize two 90-nucleotide (90-nt) baits per locus.

| **Sample** | **Code** | **Cluster** |
| --- | --- | --- |
| SR1 | SRO | Nothern Group |
| BB11 | NAY | Nothern Group |
| BB12 | NAY | Nothern Group |
| Col4 | COL | Nothern Group |
| Col5 | COL | Nothern Group |
| Mich3 | MCH | Nothern Group |
| Gro6 | GRO | Nothern Group |
| Gro7 | GRO | Nothern Group |
| Oax2 | OAX | Nothern Group |
| Oax3 | OAX | Nothern Group |
| Guat9 | GUA | Nothern Group |
| CR5 | CRI | Nothern Group |
| CR7 | CRI | Nothern Group |
| Ecu2 | ECU | Southern Group |
| Ecu4 | ECU | Southern Group |

**Table S1.** **Samples used for baits design.** The code corresponds for nine localities along the Tropical Eastern Pacific of *Lutjanus guttatus*. Santa Rosalía (SRO), Nayarit (NAY), Colima (COL), Michoacán (MCH), Guerrero (GRO), Oaxaca (OAX), Guatemala (GUA), Costa Rica (CRI), and Ecuador (ECU).

For bait design, we submitted this information to Arbor Biosciences (MI, USA) where it was analyzed in combination with other species for which species-specific baits were also designed and synthesized in one batch (i.e., *L. peru, Thunnus albacares, Coryphaena hippurus, Scomberomorus sierra, S. concolor, Sphyrna lewini, Periophthalmus modestus and Eurycea bislineata* (see Pierson *et al*., 2021). We used the sofware RepeatMasker *v*. 4.0.8 (Tarailo-Graovac and Chen, 2009) to repeat-masked against vertebrate lineage repeats (taxon: Vertebrate, engine: cross_match) and to soft-mask all repetitive elements and simple repeats. Similar sequences with at least 90% overlap and at least 95% sequence identity were collapsed into clusters using USEARCH *v*. 8.1.1.1861 (Alloui *et al*., 2015) where one representative sequence of each cluster was retained. Then 90-nt baits, two per locus (one for Read1 and one for Read2) were designed with ~20 nt flexible spacing. Then each bait candidate was blasted against five genomes that span the group of organisms considered (*Lates calcarifer:* GCF_001640805.1, *Thunnus orientalis*: GCA_009176245.1, *Rhincodon typus*: GCF_001642345.1, *Miichthys miiuy*: GCA_001593715.1, and *Nanorana parkeri: GCF_000935625.1)*. For each bait, the BLAST hit with the highest hybridization melting temperature, *Tm*, was discarded, and then the top 1000 hits (by bit score) were considered, and non-specific baits based on *Tm* were filtered out. Based on the distribution of remaining calculated *Tm*’s we then filtered out non-specific baits using the following criteria: we removed baits with repeats, and of the remaining we kept those that had at most 10 hits 62.5–65 °C and 4 hits above 65 °C, and fewer than 2 passing baits on each flank. Also, we selected baits with GC content >30% and < 60%. We kept one bait in the Read1 and one bait in the Read2 per locus, and only considered those where both, Read1 and Read2, presented baits passing the filters. This set was synthesized as an RNA custom myBaits® kit in combination with those from other species specified above by Arbor Biosciences®.

**RADcap library and sequencing molecular protocol**

We generated triple-enzyme RADseq libraries (3RAD), for 192 individuals, which were normalized to 20 ng/ µL, using 5 µL per sample for a final concentration of 100 ng. Samples were digested for 1 hr at 37 °C with the follow reaction mix, 1 µL of 10X CutSmart Buffer, 5 µL of dH_2_O, 0.5 µL of BamHI at 20 U/ µL, 1 µL of ClaI at 10 U/ µL, and 0.5 µL of MspI at 20 U/ µL, 1 µL at 5 µM of double-stranded iTru R1 adapter (Cla_I adapter) and 1 µL at 5 µM of double-stranded iTru R2 adapter (Bam_HI adapter) and 5 µL of DNA. After incubation at 37 °C, 96 different combination of internal index adapters were ligated to the samples, adding 2 µL of dH_2_O, 1.5 µL of ATP at 10 µM, 0.5 µL of 10x Ligase Buffer and 1 µL of T4 DNA Ligase (100 U/ µL) per reaction. The reaction were carried out with the following conditions: two cycles of 22 °C for 20 min and 37 °C for 10 min, followed by a single cycle of 80 °C for 20 min.

After ligation two independent pools with 96 samples were constructed, each pool was cleaned using Sera-Mag SpeedBeads (Thermo Fisher Scientific, Waltham, MA, USA), at a ratio of 1.2:1 SpeedBeads-DNA, the cleaned product was resuspended in 20 µL of IDTE buffer. A single-cycle PCR was carried out for generate the full-length library construction, using 5 µL of Kapa HiFi Buffer (Roche, Basel, Switzerland), 0.5 µL of dNTPs at 10 µM, 8.75 µL of H_2_O, 0.5 µL of Kapa HiFi DNA Polymerase at 1 U/µL, 2.5 µL of iTru 7 at 5 µM and 2.5 µL at 5 µM of a degenerate iTru5 primer (with 8N) for downstream filtering of PCR duplicates. The thermocycler condition were: 95 °C for 2 min, followed by 14 cycles of 98 °C for 20 sec., 60 °C for 15 sec., 72 °C for 30 sec., and finally 72 °C for 5 min. After PCR, 5 µL for this product were ran in a 1.5% agarose gel for 45 min at 90 volts, the library was considered successful when a smear of evenly distributed and bright DNA around ~300-800 bp was observed. After evaluation the PCR products were cleaned with SpeedBeads in a ratio of 1.2:1 (SpeedBeads-DNA), the cleaned products were eluted in 20 µL of IDTE buffer.

The final products were quantified using a Qubit Fluorimeter (Thermo Fisher Scientific, Waltham, MA, USA). The pools concentrations were of 10 ng/µL in a final volume of 60 µL. Two pools were size-selected using a Pippin Prep (Sage Science, Beverly, MA, USA) with a 1.5% dye-free Marker K agarose gel cassette (CDF1510) set to capture fragments at 550 +/- 10%. Three limited-cycle PCR P5 and P7 primers were performed to increase the concentration (for detailed description see Hoffberg *et al*., 2016; Glenn *et al*., 2019), with the following conditions 3 cycles of 98 °C for 20 sec, 60 °C for 15 sec., 72 °C for 30 sec.; followed by a single cycle of 72 °C for 5 min. This product was cleaned using the SpeedBeads with a ratio ≥ 1:1 (SpeedBeads-DNA), and the DNA was resuspended in a 25 IDTE buffer.

Capture process for the two pools were realized following the manufacturer's protocol (MYcroarray MYbaits v3.0 protocol) and the complete process was made at the Environmental Health Science department at the University of Georgia, using temperatures between 62.5 °C to 65 °C, selecting baits with GC content >30% and < 60%. Finally, we kept one bait for Read1 and Read2, and those that passed the filters. This set of baits were synthesized as an RNA custom myBaits® kit.

**Supplementary References**

**Alloui T, Boussebouhg I, Chaoui A, Nouar AZ, Chettah MC.** 2015. “Usearch: A meta search engine based on a new result merging strategy”, in 2015 7^th^ International Joint Conference on Knowledge Discovery. Knowledge Engineering and Knowledge Management (IC3K): IEEE, November 12, 2015 531-536.

**Bayona-Vásquez NJ, Glenn TC, Kieran TJ, Pierson TW, Hoffberg SL, Scott PA, Bentley KE, Finger JW, Louha S, Troendle N, et al.** 2019. Adaptarema III: quadruple-indexed, double/triple-enzyme RADseq libraries (2RAD/3RAD). *PeerJ* 7 e7724.

**Catchen J, Amores A, Hohenlohe P, Cresko W, Postlethwait JH.** 2011. Stacks: building and genotyping loci *de novo* from short-read sequences G3: Genes, Genomes, Genet. 1 (3): 171-182.

**Catchen J, Hohenlohe, Bassham S, Amores A, Cresko WA.** 2013. Stacks: an analysis tool set for population genomics. Mol. Ecol. 22 (11): 3124-3140.

**Glenn TC, Pierson TW, Bayona-Vásquez NJ, et al**. 2019. Adaptarema II: Universal amplicon sequencing on Illumina plataforms (TaggiMatrix). *PeerJ* 7:e7786. <https://doi.org/10.7717/peerj.7786>

**Hoffberg SL, Kieran TJ, Catchen JM et al.**, 2016. RADcap: sequence capture of dual-digest RADseq libraries with identificable duplicates and reduced missing data Mol. Ecol. Res 16: 1264-1278.

**Pàges H, Aboyoun P, Gentleman R, DebRoy S.** 2019. Biostrings: Efficient manipulation of biological strings. Retrieved from <https://bioconductor.org/packages/release/bioc/html/Biostrings.html>.

**Pierson TW, Fitzpatrick BM, Camp CD.** 2021. Genetic data reveal fine-scale ecological segregation between larval plethodontid salamanders in replicate contact zones. Evolutionary Ecology. 35: 309-322.

**Rice P, Longden I, Bleasby A.** 2000. EMBOSS: the European molecular biology open software suite. Trends Genet. 16: 276-277.

**Tarailo-Graovac M, Chen N**. 2009. Using RepeatMasker to identify repetitive elements in genomics sequences. *Curr Protoc Bioinforma*. Chapter 4, Unit 4.10.
