# Supplementary material for: Genomic assessment reveals signal of adaptive selection in populations of the Spotted rose snapper Lutjanus guttatus from the Tropical Eastern Pacific
Source: PeerJ. 2023 Mar 27;11:e15029. doi: 10.7717/peerj.15029 (PMC10062342; doi:10.7717/peerj.15029)
Supplement: File S2 — Results for the 2003 SNPs corresponding to the combined loci (CL) dataset. [file peerj-11-15029-s011.docx]

**File S2**

**Genomic assessment reveals signal of adaptive selection in populations of the Spotted rose snapper *Lutjanus guttatus* from the Tropical Eastern Pacific**

Adan Fernando Mar-Silva^1^, Pindaro Díaz-Jaimes^2^, Cristina A. Dominguez-Mendoza^2^, Omar Domínguez-Domínguez^3,4^, Jonathan Valdiviezo-Rivera^4^ and Eduardo Espinoza^5^

**Supplementary results for the 2003 SNPs that correspond with the combined loci (CL) dataset.**

**Genetic diversity**

We observed positive *F_IS_* values and values of confidence intervals which suggest some degree of deficiency in heterozygosity as all results are above zero. The localities of OAX, LPA, and SRO were excluded of these analyses due to small sampling size (1, 3 and 3 samples per locality respectively) (Fig. 1C, File S1). The range values per locality also show a similar value for H_O_ (0.048 to 0.085 for SRO and MCH), H_e_ (0.058 to 0.126 for LPA and COL) and for Ar (1.057 to 1.122 for SRO and COL) (Fig. 1A, 1B and 1D).


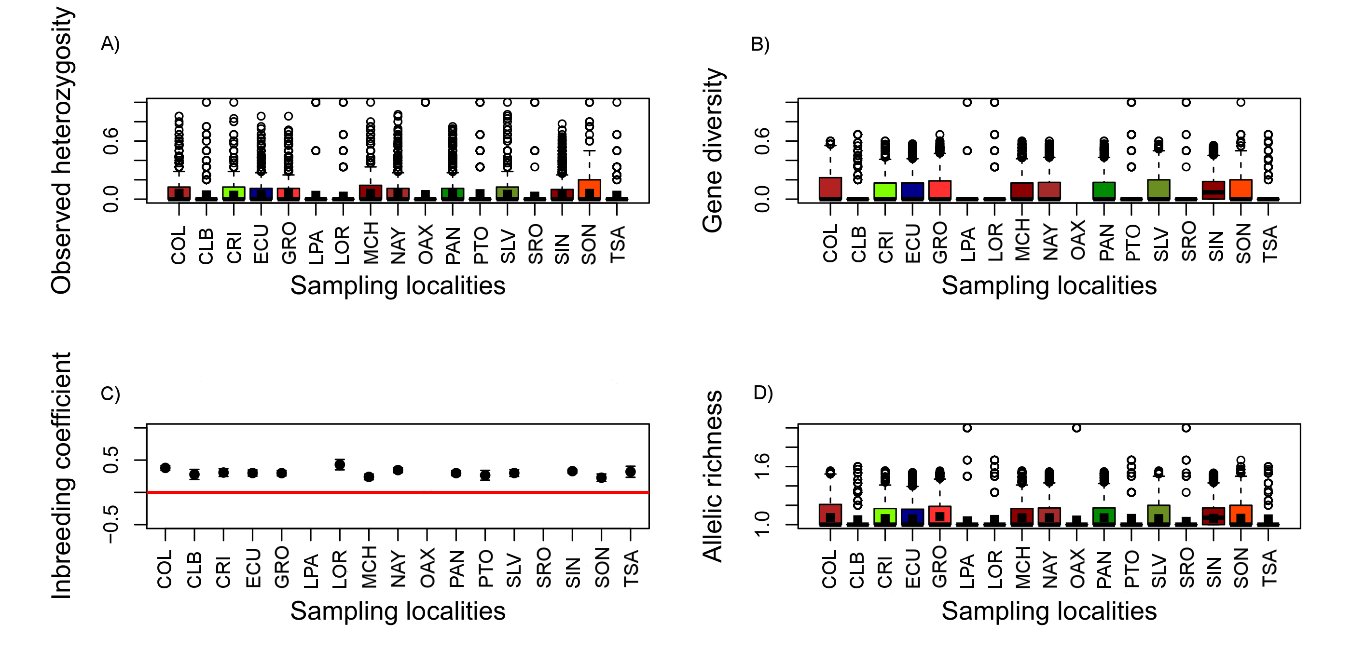


**Fig 1.** Genetic diversity indices for CL based in 2003 SNPs for *Lutjanus guttatus*. A) Observed heterozygosity (H_O_), B) expected heterozygosity (H_e_), C) inbreeding coefficient (F_IS_), D) Allelic richness (Ar).

**Population genetic structure**

Pairwise *F_ST_* estimated with the CL dataset showed significant values of differentiation in three localities, PAN (*F_ST_* range 0.019, *p* = 0.040 to 0.065), CLB (*F_ST_* range 0.038, *p* = 0.018 to 0.120, *p* = 0.018) and ECU (*F_ST_* range 0.054, *p* = 0.0001 to 0.091, *p* = 0.0001), which represent the southern region of the specie’s distribution (Fig. 1, main manuscript). Contrary, localities from Baja California (*e.g*., SRO, LOR, LPA and TSA), did not present differentiation with the Southern populations (Table 1).

SAMOVA analysis for combined loci (CL) suggest that the best scheme of the genetic structure of *Lutjanus guttatus* is represented by two groups (K = 2) (*F_CT_* = 0.0653; *p* < 0.001), which maximize the variation between populations. These groups are composed by the localities of SRO, SON, LOR, LPA, TSA, SIN, NAY, COL, MCH, GRO, PTO, OAX, SLV, and CRI, which correspond to Northern localities of the species; and PAN, CLB, and ECU, that are part of the Southern region (Table 2).

**Table 2.** Results of SAMOVA analysis for the CL dataset (2003 SNPs). The better explanation for the recovered clusters was represented without defined groups.

| **Two groups** | **Source of variation** | **% of variance** | **Fixatio index** | ***p-*value** |
| --- | --- | --- | --- | --- |
| [SRO-SON-LOR-LPA-TSA-SIN-NAY-COL-MCH-GRO-PTO-OAX-SLV-CRI]  +  [PAN-CLB-ECU] | Among groups | 6.54 | *F****_CT_* = 0.0653** | < 0.001 |
|  | Among populations within groups | -2.3 | *F****_SC_* = -0.0246** | NS |
|  | Among individuals whithin population | 4.61 | *F****_IS_* = 0.0480** | < 0.001 |
|  | Whitin individuals | 91.16 | *F****_IT_* = 0.0884** | < 0.001 |

**Note:** NS = not significant

The hierarchical AMOVA was conducted using the clusters defined by the SAMOVA by evaluating two groups (Northern and Southern populations). The AMOVA hypothesis evaluated for CL showed significant genetic differences for intra-group variance (*F_CT_ =* 0.0553; *p* = 0.001) for the Northern and Southern groups (Table 3).

**Table 1.** Pairwise *F_ST_* values for CL dataset for *Lutjanus guttatus*.

|  | **SRO** | **SON** | **LOR** | **LPA** | **TSA** | **SIN** | **NAY** | **COL** | **MCH** | **GRO** | **PTO** | **OAX** | **SLV** | **CRI** | **PAN** | **CLB** | **ECU** |
| --- | --- | --- | --- | --- | --- | --- | --- | --- | --- | --- | --- | --- | --- | --- | --- | --- | --- |
| **SRO** | 0 | 0.999 | 0.999 | 0.601 | 0.965 | 0.999 | 0.999 | 0.999 | 0.999 | 0.999 | 0.999 | 0.999 | 0.999 | 0.999 | 0.999 | 0.967 | 0.999 |
| **SON** | -0.637 | 0 | 0.565 | 0.814 | 0.871 | 0.334 | 0.313 | 0.472 | 0.683 | 0.071 | **0.037** | 0.999 | **0.047** | 0.109 | **0.000** | **0.008** | **0.000** |
| **LOR** | -0.820 | -0.009 | 0 | 0.695 | 0.519 | 0.855 | 0.977 | 0.940 | 0.986 | 0.871 | 0.594 | 0.999 | 0.622 | 0.129 | 0.106 | 0.089 | **0.044** |
| **LPA** | 0.011 | -0.050 | -0.003 | 0 | 0.436 | 0.969 | 0.952 | 0.916 | 0.913 | 0.949 | 0.303 | 0.999 | 0.867 | 0.612 | 0.885 | 0.713 | 0.877 |
| **TSA** | -0.438 | -0.049 | 0.008 | 0.046 | 0 | 0.995 | 0.982 | 0.988 | 0.970 | 0.972 | 0.413 | 0.999 | 0.806 | 0.717 | 0.535 | 0.081 | 0.325 |
| **SIN** | -0.619 | 0.011 | -0.012 | -0.133 | -0.074 | 0 | 0.293 | 0.880 | 0.980 | 0.178 | 0.627 | 0.999 | 0.140 | 0.965 | **0.000** | **0.030** | **0.000** |
| **NAY** | -0.568 | 0.008 | -0.033 | -0.080 | -0.058 | 0.008 | 0 | 0.726 | 0.625 | 0.393 | 0.152 | 0.999 | 0.084 | 0.997 | **0.000** | **0.018** | **0.000** |
| **COL** | -0.671 | 0.001 | -0.021 | -0.079 | -0.067 | -0.003 | -0.001 | 0 | 0.669 | 0.311 | 0.552 | 0.999 | 0.160 | 0.913 | **0.000** | **0.007** | **0.000** |
| **MCH** | -0.621 | -0.006 | -0.036 | -0.145 | -0.058 | -0.010 | 0.001 | 0.000 | 0 | 0.293 | 0.618 | 0.999 | 0.067 | 0.832 | **0.000** | **0.040** | **0.000** |
| **GRO** | -0.515 | 0.016 | -0.021 | -0.117 | -0.061 | 0.010 | 0.000 | 0.005 | 0.007 | 0 | 0.229 | 0.999 | 0.136 | 0.993 | **0.000** | **0.025** | **0.000** |
| **PTO** | -0.665 | **0.039** | 0.019 | 0.023 | 0.011 | 0.005 | 0.030 | 0.006 | 0.001 | 0.020 | 0 | 0.999 | 0.171 | **0.044** | **0.004** | **0.018** | **0.029** |
| **OAX** | -0.737 | -0.170 | -0.145 | 0.155 | -0.053 | -0.158 | -0.173 | -0.172 | -0.212 | -0.112 | 0.009 | 0 | 0.999 | 0.501 | 0.539 | 0.505 | 0.713 |
| **SLV** | -0.540 | **0.029** | 0.000 | -0.089 | -0.018 | 0.017 | 0.018 | 0.014 | 0.025 | 0.013 | 0.038 | -0.114 | 0 | 0.631 | **0.000** | **0.016** | **0.000** |
| **CRI** | -0.443 | 0.025 | 0.048 | 0.000 | -0.016 | -0.018 | -0.044 | -0.018 | -0.011 | -0.046 | **0.065** | 0.017 | -0.001 | 0 | **0.040** | **0.004** | 0.079 |
| **PAN** | -0.516 | **0.062** | 0.030 | -0.089 | 0.000 | **0.053** | **0.048** | **0.055** | **0.042** | **0.050** | **0.056** | -0.022 | **0.065** | **0.019** | 0 | 0.431 | 0.438 |
| **CLB** | -0.272 | **0.098** | 0.063 | 0.001 | 0.069 | **0.040** | **0.038** | **0.046** | **0.041** | **0.034** | **0.120** | 0.032 | **0.052** | **0.066** | 0.001 | 0 | 0.342 |
| **ECU** | -0.558 | **0.091** | **0.061** | -0.079 | 0.013 | **0.057** | **0.057** | **0.055** | **0.054** | **0.058** | **0.070** | -0.037 | **0.055** | 0.021 | 0.002 | 0.009 | 0 |

**Notes:** Significant values are highlighted in bold, *p-*values are presented above the diagonal. SRO: Santa Rosalía, SON: Sonora, LOR: Loreto, LPA: La Paz, TSA: Todos Santos, SIN: Sinaloa, NAY: Nayarit, COL: Colima, MCH: Michoacán, GRO: Guerrero, PTO: Partidito, OAX: Oaxaca, SLV: Salvador, CRI: Costa Rica, PAN: Panamá, CLB: Colombia, ECU: Ecuador

**Table 3.** Results of AMOVA for the Combined Loci (CL) dataset of *Lutjanus guttatus* considering the two groups (Northern and Southern) obtained in the SAMOVA.

| **Two groups** | **Source of variation** | **% of variance** | **Fixation index** | ***p-*value** |
| --- | --- | --- | --- | --- |
| Northern  +  Southern | Among groups | 5.53 | *F_CT_* = 0.0553 | 0.001 |
|  | Among populations within groups | -2.3 | *F_SC_* = -0.0243 | NS |
|  | Within populations | 96.77 | *F_ST_* = 0.0323 | NS |

**Note:** NS = not significant

DAPC analyses identified Northern and Southern groups as main clusters, which were also defined by SAMOVA, and subsequently evaluated by AMOVA (Fig 2A). In addition, when the DAPC was perform defining groups, both clusters were recovered (Fig 2B).


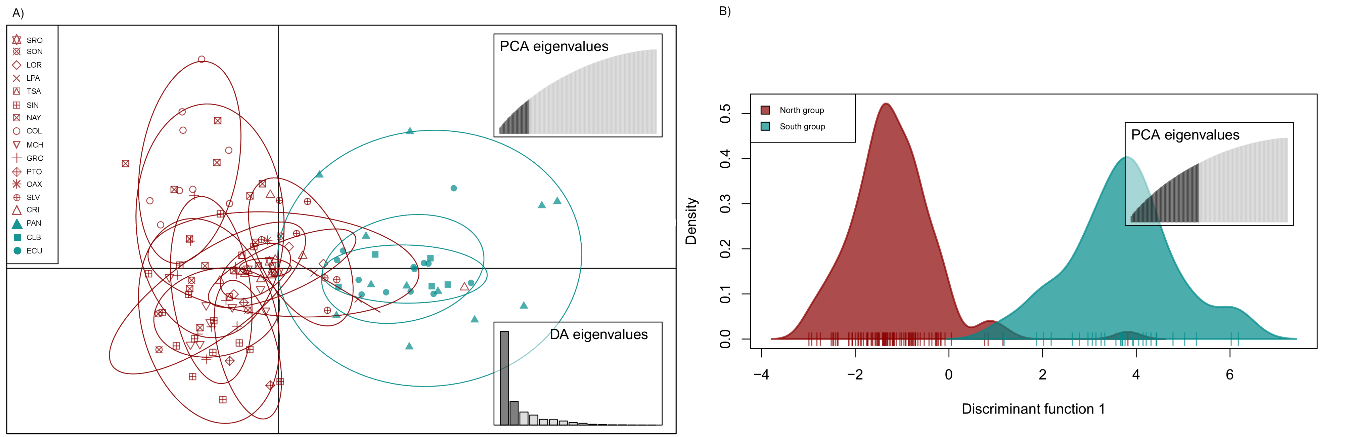


**Fig 2.** Discriminant Analysis of Principal Component (DAPC) analysis based in 2003 SNPs. A) Non previous grouping, versus B) *a priori* groups.

The Admixture analysis suggest two well defined clusters (K = 2) that represents the better explanation for the genetic variation of the populations (Fig. 3). This analysis suggests that the Northern group, is conformed with the localities of SRO, LOR, LPA TSA, SIN, NAY, COL SON, MCH, GRO, OAX, SLV, and CRI0. While, the Southern group showed a clear differentiation between the localities of CLB, ECU, and PAN (Fig. 4).


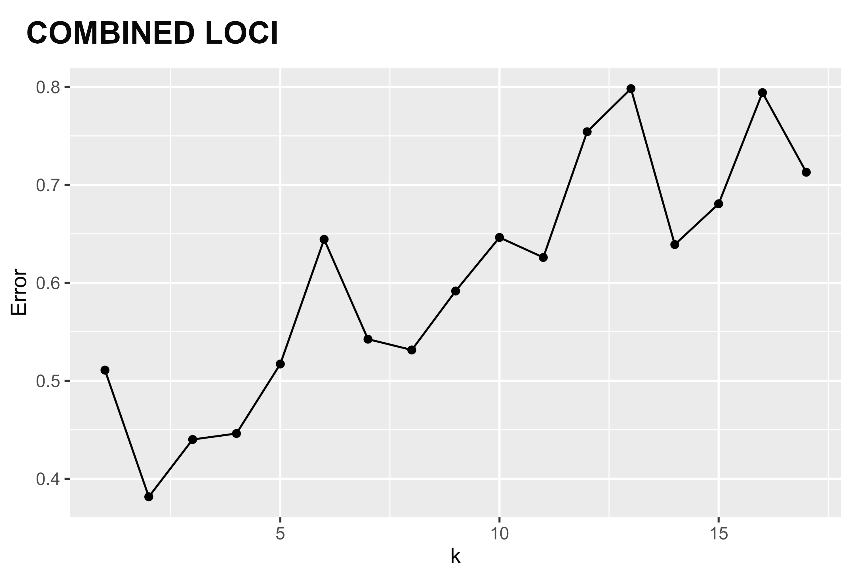


**Fig 3.** Cross-Validation Error (CVE) based in 2003 SNPs (CL dataset) obtained with ADMIXTURE.


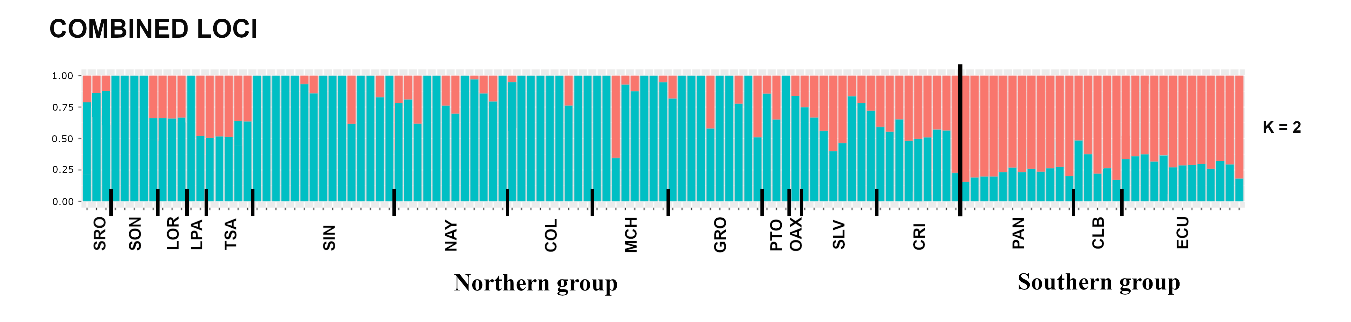


**Fig 4.** Admixture analysis for the 17 localities of *Lutjanus guttatus* based in 2003 SNPs (CL dataset). Each individual is represented by a bar; colors represent the inferred membership of each K (2). Each locality and its respective cluster are indicated at the bottom of the figure.

**IBD, IBE and IBR**

The IBD showed no significant correlation between *F_ST_* and the Euclidean geographic distance for *L. guttatus* (r = 0.1101, *p* = 0.1945) (Fig. 5).


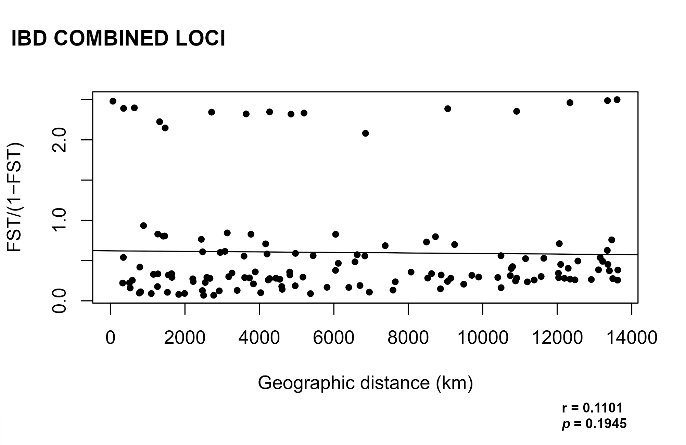


**Fig. 5.** IBD results using a mantel test for *Lutjanus guttatus*. Geographical distance was transformed to natural logarithm (ln).

Contrary, the IBE and IBR showed significant correlation between the *F_ST_* and the environment, as well as with the current velocity (r = 0.2459; *p* = 0.0101 and r = 0.3687; *p* = 0.0012, respectively) (Fig. 6).


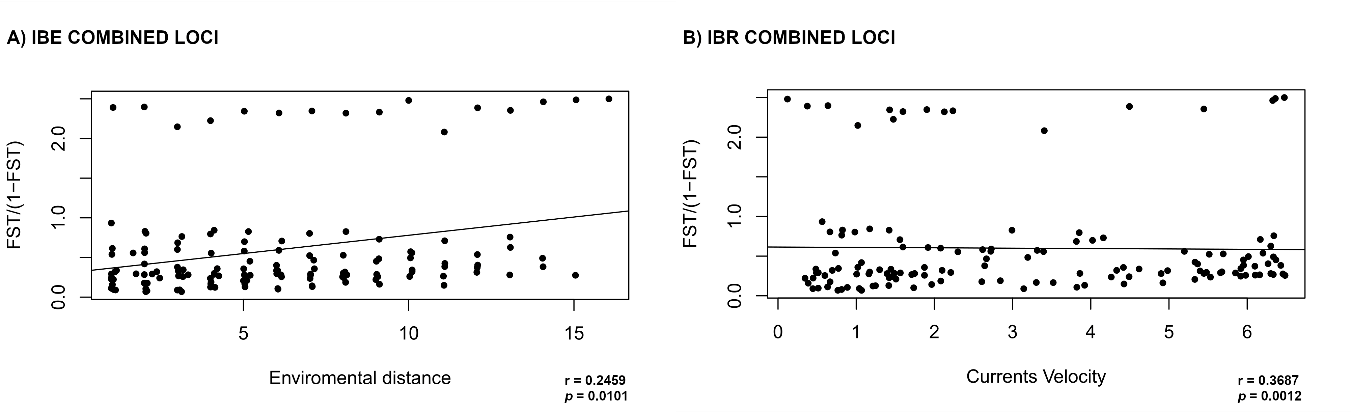


**Fig. 6.** A) IBE and B) IBR for *Lutjanus guttatus*. Five variables were used for the IBE (temperature, salinity, primary production, phytoplankton and chlorophyll), and the current velocity for IBR

**Spatial structure and genetic diversity**

The EEMS analysis identified a possible barrier to gene flow between the Northern and the Southern groups, in the region of the Gulf of Panama, which is influenced by the Panama Bight Gyre (Fig 7). These analyses also suggest a barrier to population connectivity off the coast in the Gulf of California, which could play an important role on the limited migration of individuals from the localities of the Gulf of California into the Mexican coast localities (NAY, COL, MCH, and GRO) (Fig. 7). The observed migration rates near to the shoreline seem to be limited, which could be related to the null movement of adults, while our data suggest a corridor found off the coast, possibly related to the Costa Rica Coastal Current, which reinforces the hypothesis that the movement of individuals is mediated during their larval stage (Fig 7).

Genetic diversity estimated by the EEMS showed high diversity rates for the Northern cluster in the localities of SRO, SON, LOR, LPA, (Fig. 8). For the Northern group the lower diversity was observed in CRI and SLV localities, while the highest was found in the localities of the Gulf of California. For the Southern group highest value was found in PAN, and the lower in ECU (Fig. 8).


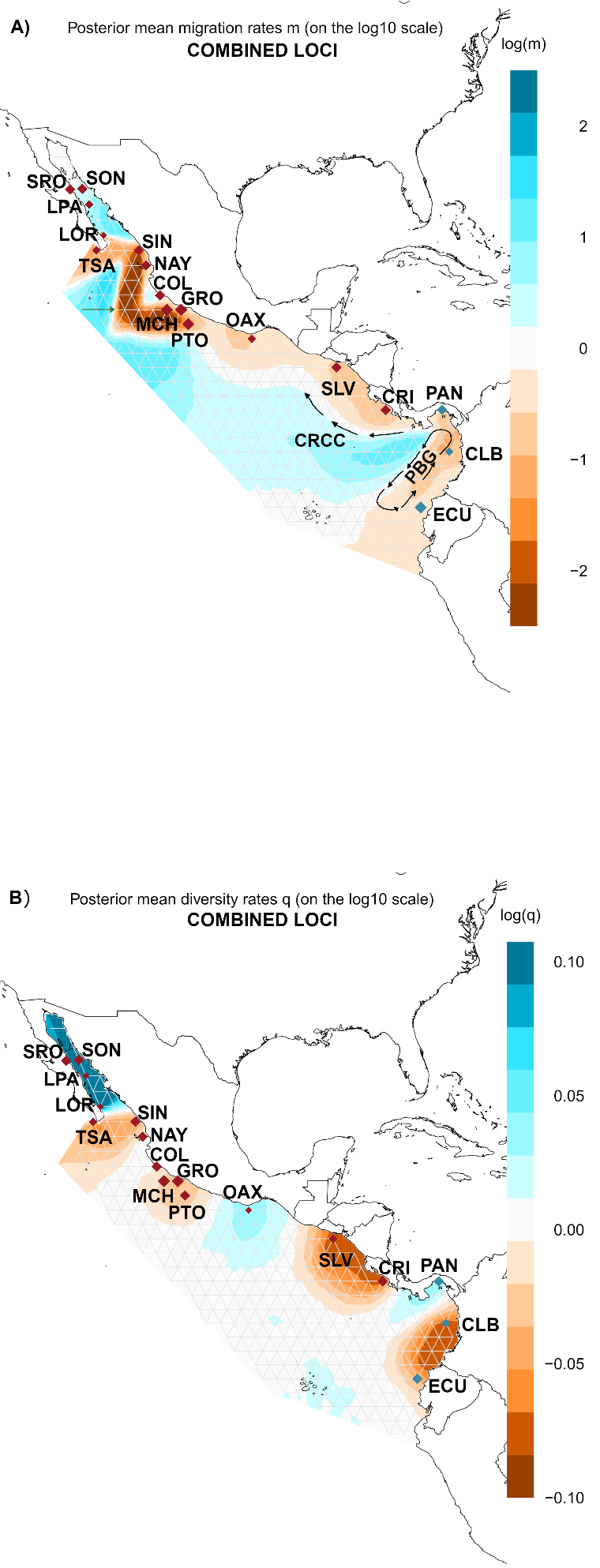


**Fig. 7.** Model of Estimated Effective Migration Surfaces (EEMS). Migration rate (m), in blue are indicated values where the migration is higher than average. In brown the migration is lower than average (the green arrow indicates barrier for the gene flow across populations). PGB: Panama Bight Gyre. B), CRCC: Costa Rica Coastal Current

**
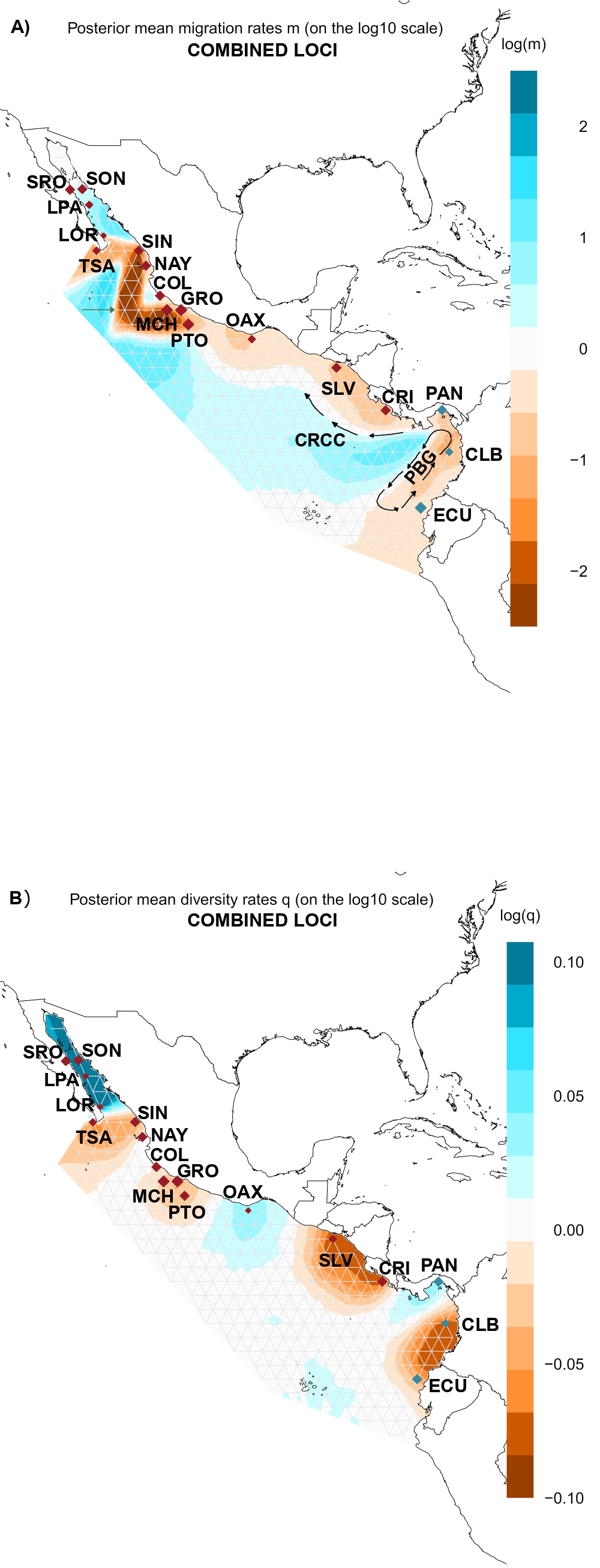
**

**Fig. 8**. Genetic diversity rate calculated with the EEMS, in blue are indicated values of high diversity, in brown values of low diversity.
